# Postanoxic Burst Suppression Electroencephalogram in a Comatose Child Associated with Spontaneous Eyelid Opening

**DOI:** 10.1155/2012/760407

**Published:** 2012-01-04

**Authors:** John R. Crawford

**Affiliations:** The Department of Neurosciences and Pediatrics, Rady Children's Hospital, University of California, San Diego, CA 92123, USA

## Abstract

Spontaneous eye opening associated with burst suppression electroencephalogram has been reported in adults following postanoxic injury. Previous reports have correlated the onset of epileptiform bursts with the eye opening and attribute it to a brainstem-release phenomenon associated with poor prognosis. The author presents a case of a 12-year-old boy with burst suppression electroencephalogram following severe anoxic injury where the eye opening occurred at the conclusion of the bursts that has never been previously reported. These electroencephalographic findings are important for intensive care physicians to recognize and may provide further insight into the pathophysiological mechanism of this rare phenomenon.

## 1. Case Report

**Figure 1:**
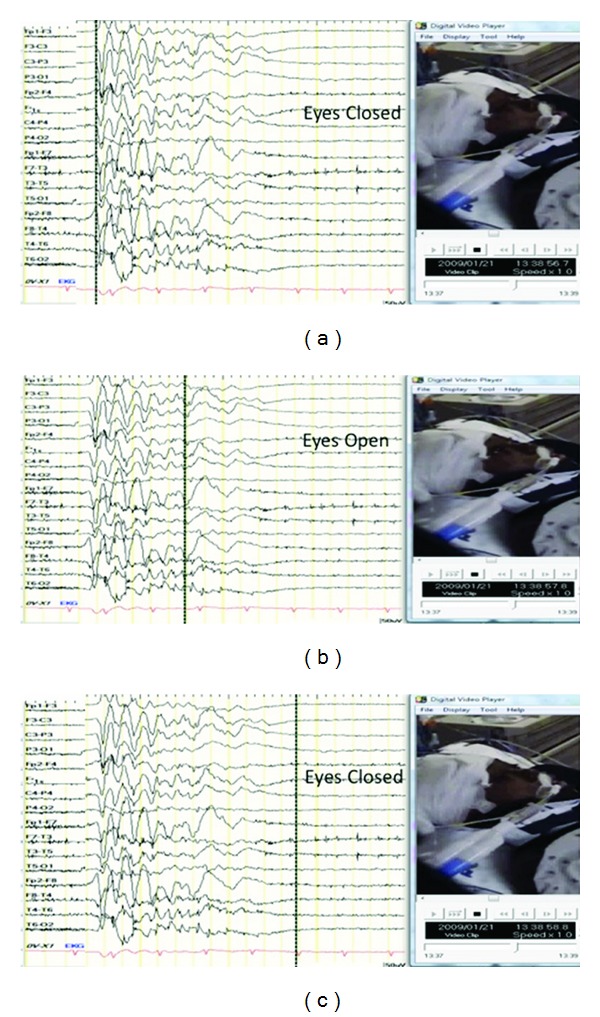
Burst suppression electroencephalogram associated with spontaneous eyelid opening. Electroencephalogram reveals burst suppression pattern with eyelid closure coinciding with onset of suppression (a, c). Eyelid opening was observed following 2 seconds of generalized bursts (b), was not mechanically suppressible, and did not cease following anticonvulsant therapy. Dashed line on the electroencephalogram recording corresponds to the initiation of eyelid movements.

A 12-year-old previously healthy boy was found in cardiopulmonary arrest following prolonged smoke inhalation. Neurologic examination revealed a comatose child with minimally reactive pupils, flaccid tone, and diffuse areflexia. Post-resuscitation he was placed on continuous electroencephalographic monitoring where he was observed to have periodic eyelid opening occurring at 20-second intervals in the absence of other movements. The eyelid opening did not coincide with onset of generalized bursts (Figure 1), as has been reported in similar patients [1–5]. Following intravenous anticonvulsant therapy, eyelid opening did not cease, the electroencephalogram eventually became isoelectric, and the patient died within 48 hours of initial insult.

## 2. Discussion

Spontaneous eyelid opening reported in posthypoxic comatose patients associated with burst suppression electroencephalogram has been reported in adults following post-anoxic injury. The presumptive mechanism is believed to arise from a pathological activation of the oculomotor nuclear complex resulting in bilateral contraction of the levator palpebrae superioris muscles via the superior division of the oculomotor nerve. A recently reported case series of 4 adult patients with similar electroencephalogram findings demonstrated that they eyelid opening corresponded to the initiation of epileptiform bursts, suggesting it is the bursts that drives the levator palpebrae superioris activity [5]. Since these findings are not observed in anesthesia-induced burst suppression, they are most likely a release phenomenon due to diffuse hypoxic-ischemic injury. However, the timing of the epileptiform bursts and eyelid opening may provide additional insight into pathologic mechanisms of activation as this has not been previously reported. It is very possible this phenomenon is on the same spectrum as post hypoxic myoclonus given the association with hypoxic injury and the periodic timing of the movements. Independent of the timing of the bursts, this phenomenon is associated with poor prognosis as has been previously reported in a larger series of adult patients [5]. This rare electroencephalographic finding is important for both child and adult intensivists and neurologists to recognize for both prognostic implications as well as to educate the families of the nonpurposeful nature of the periodic eye opening postsevere hypoxic ischemic injury.

## Supplementary Material

Video demonstrates a continuous recording of a burst suppression electroencephalogram associated with periodic movements of eyelid opening. As demonstrated in the video, eyelid opening occurred at the near conclusion of the epileptiform bursts and was not suppressible with anticonvulsant therapy.
